# Initial mycophenolate dose in tacrolimus treated renal transplant recipients, a cohort study comparing leukopaenia, rejection and long-term graft function

**DOI:** 10.1038/s41598-020-76379-6

**Published:** 2020-11-09

**Authors:** Vatsa Dave, Kevan R. Polkinghorne, Khai Gene Leong, John Kanellis, William R. Mulley

**Affiliations:** 1 Department of Nephrology, Monash Medical Centre, 246 Clayton Road, Clayton, VIC 3168 Australia; 2 Department of Medicine, Centre for Inflammatory Diseases, Monash University, Clayton, VIC 3168 Australia; 3 Department of Epidemiology and Preventive Medicine, Monash University, Prahran, VIC Australia

**Keywords:** Immunology, Nephrology

## Abstract

The evidence supporting an initial mycophenolate mofetil (MMF) dose of 2 g daily in tacrolimus-treated renal transplant recipients is limited. In a non-contemporaneous single-centre cohort study we compared the incidence of leukopaenia, rejection and graft dysfunction in patients initiated on MMF 1.5 g and 2 g daily. Baseline characteristics and tacrolimus trough levels were similar by MMF group. MMF doses became equivalent between groups by 12-months post-transplant, driven by dose reductions in the 2 g group. Leukopaenia occurred in 42.4% of patients by 12-months post-transplant. MMF 2 g was associated with a 1.80-fold increased risk of leukopaenia compared to 1.5 g. Rejection occurred in 44.8% of patients by 12-months post-transplantation. MMF 2 g was associated with half the risk of rejection relative to MMF 1.5 g. Over the first 7-years post-transplantation there was no difference in renal function between groups. Additionally, the development of leukopaenia or rejection did not result in reduced renal function at 7-years post-transplant. Leukopaenia was not associated with an increased incidence of serious infections or rejection. This study demonstrates the initial MMF dose has implications for the incidence of leukopaenia and rejection. Since neither dose produced superior long-term graft function, clinical equipoise remains regarding the optimal initial mycophenolate dose in tacrolimus-treated renal transplant recipients.

## Introduction

The combination of tacrolimus, MMF and prednisolone is commonly used for de novo renal transplant recipients and has been associated with excellent clinical outcomes relative to other regimens^1^. The recommended starting dose of MMF for renal transplantation is 2 g daily. This is based on initial dose finding studies in cyclosporine treated patients^2,3^. It is well established that mycophenolic acid (MPA) exposure is substantially higher with tacrolimus than with cyclosporine, predominantly due to less inhibition of enterohepatic recirculation of MPA with tacrolimus^4–6^. Therefore, it is possible that lower doses of MMF may be sufficient in tacrolimus treated patients. However, most but not all studies examining 1 g of MMF daily compared with 2 g daily in tacrolimus treated patients, found an increase in the incidence of rejection with the lower dose^7–10^.

To the best of our knowledge there have been no studies directly comparing the efficacy and adverse effects of an intermediate MMF starting dose of 1.5 g daily compared with 2 g daily over the long-term. Given that MMF is associated with considerable side-effects including gastrointestinal upset, bone marrow suppression and non-specific effects of immunosuppression such as malignancy and infection, limiting drug exposure would seem a worthy goal^11^.

We hypothesised that 1.5 g of MMF daily would have efficacy equivalent to 2 g daily and that the higher dose would be associated with more adverse events. We focused on the outcomes of rejection for efficacy and leukopaenia for adverse events and additionally examined the impact of these 2 dosing regimens on long-term graft function.

## Patients and methods

### Study design and patients

The study has a retrospective non-contemporaneous cohort design. Consecutive adult (age ≥ 18 years) kidney transplant and simultaneous pancreas and kidney transplant recipients who underwent de novo transplantation at Monash Medical Centre during the period 1st of April 2008 until the 28th of February 2011 were included. This period was chosen as it straddled the time-point (October 2009) at which our centre changed the initial standard starting dose of MMF used with tacrolimus from 1.5 g daily to 2 g daily and allowed similar numbers of patients in each group. Patients commenced on maintenance immunosuppressive regimens other than tacrolimus, MMF and prednisolone were excluded. MMF was administered as 1 g twice daily (2 g daily group) or 750 mg twice daily (1.5 g daily group), mycophenolate sodium was used in a minority of patients with 720 mg considered equivalent to 1 g of MMF. Tacrolimus was administered orally with a single loading dose of 0.1 mg/kg followed by a maintenance dose of 0.075 mg/kg twice daily adjusted on the basis of trough levels to target 8–12 ng/mL for months 0–3, 6–10 ng/mL for 3–6 months and thence 4–8 ng/mL. Prednisolone was commenced at 20 mg daily and weaned toward 5 mg maintenance from 3 months post-transplant. All patients received basiliximab (20 mg on post-transplant days 0 and 4) induction therapy along with pulse methylprednisolone.

As the study did not require participation and all data were collected retrospectively, this study was approved as a non-interventional audit, thereby waiving the need individual patient consent, by the Monash Health Human Research Ethics Committee (Reference Number: RES-17-0000-641Q). The study was conducted in accordance with Australian ethical standards and privacy regulations.

### Other medications

For infection prophylaxis, all patients received sulphamethoxazole/trimethoprim (800 mg/160 mg) twice weekly. Patients allergic to this medication received dapsone 100 mg three times weekly. All patients received oral valganciclovir, dose-adjusted for eGFR, excepting CMV negative recipients with CMV negative donors who received oral valaciclovir adjusted for eGFR. Cellular rejection episodes were treated with pulse methyl-prednisolone and increased baseline immunosuppression where necessary. Acute antibody mediated rejection was treated as previously described^12,13^. Patients received pulse methylprednisolone for 3 days along with 4 weeks of plasma exchange (3 sessions weekly). A repeat biopsy was then undertaken with further plasma exchange provided for patients with ongoing rejection, followed by high dose intravenous immunoglobulin (3 g/kg) and rituximab (500 mg single fixed dose) in refractory and chronic cases.

### Outcome measures

The primary outcome measures were the presence of and time to the first acute rejection episode and leukopaenia episode within the first 12 months post-transplantation and long-term renal function (eGFR at 7-years). Secondary outcomes included leukopaenia duration, physician response to leukopaenia, neutropaenia, CMV viraemia, BK viraemia and hospitalisation due to infection within the first 12 months post-transplantation. Graft and patient survival were compared to 7 years post-transplant for all patients.

Leukopaenia was defined as a total white blood cell count < 4 × 10^9^ cells/L whilst neutropaenia was defined as < 2 × 10^9^ cells/L. All rejection episodes were biopsy proven and scored using Banff criteria. For standard immunological risk transplants, surveillance biopsies were performed at months 3 and 12 post-transplant and additionally as clinically indicated. For increased immunological risk transplants (ABO incompatible or donor specific antibody positive transplants) pre-emptive antibody removal with plasma exchange was undertaken and biopsies were performed 7–14 days post-transplant and then as for standard immunological risk transplants.

### Statistical analysis

Comparisons between groups were performed using the student t-test for parametric data and the Mann Whitney U-test for non-parametric data. The Chi-square test was used for comparison of proportions. Time-to-event comparisons were made using the log-rank test for unadjusted models and Cox proportional hazards for multivariable models, there was no censoring for death as there were no deaths within the included patients during the first 12 months post-transplant. Multivariable models included age, sex, rejection and leukopaenia as well as clinically relevant parameters which were significantly associated with the outcome measure on univariate analysis. The proportional hazards assumption was assessed by graphical and formal testing of Schoenfeld residuals. For the time to leukopaenia multivariate model, non-proportional hazards were seen in CMV IgG donor positive to recipient negative recipients (p = 0.002, global test p = 0.03) with a change in the risk of leukopaenia detected at 4 months post-transplant. Hence this was modelled in a time-dependent fashion (formal testing of Schoenfeld residuals p-value > 0.10 individual parameters, global test p = 0.44). For all other models the proportional hazards assumption was met both graphically and with formal testing (p-values all > 0.20). Royston–Parmar flexible parametric models were fit to model the smoothed adjusted hazard rate for leukopaenia by MMF group^14^. All analyses comparing estimated glomerular filtration rate (eGFR) and tacrolimus drug levels between groups were performed using a mixed linear model. eGFR was calculated using the CKD-Epi equation, with additional sensitivity analyses performed using the MDRD 4-variable and the Mayo Clinic quadratic equations^15–17^. Analyses were performed using Stata 15 (StataCorp, College Station, TX, USA).

## Results

**Figure 1:**
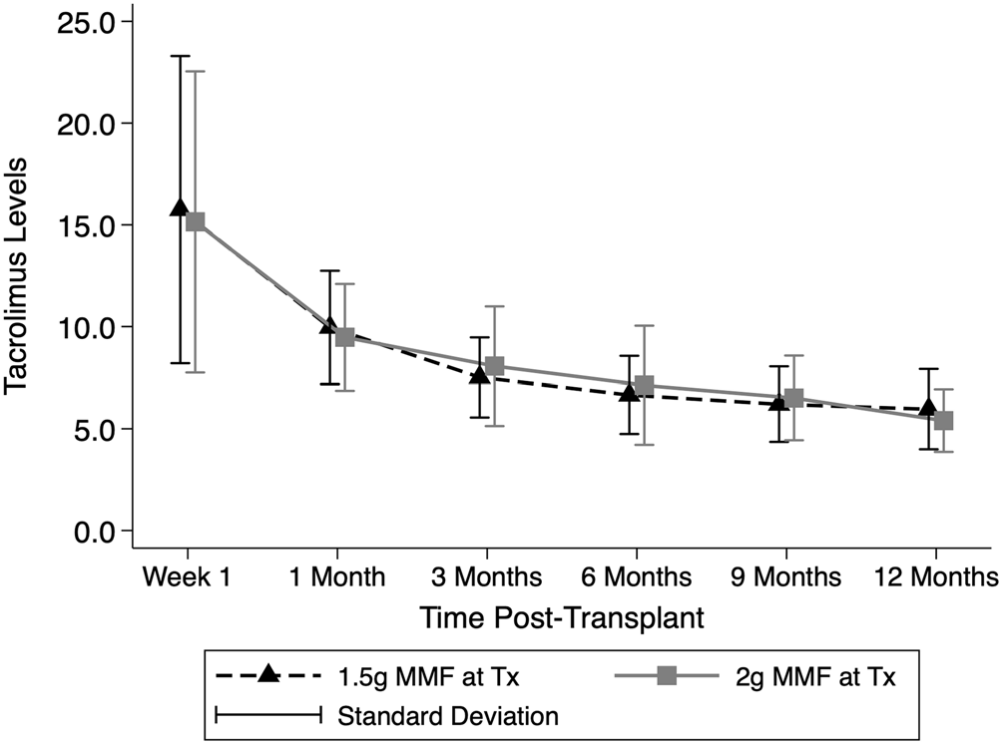
Tacrolimus levels over the initial 12 months post-transplant by mycophenolate dose group. Tacrolimus levels are in ng/mL. *MMF* mycophenolate, *g* grams, *Tx* transplant.

**Figure 2:**
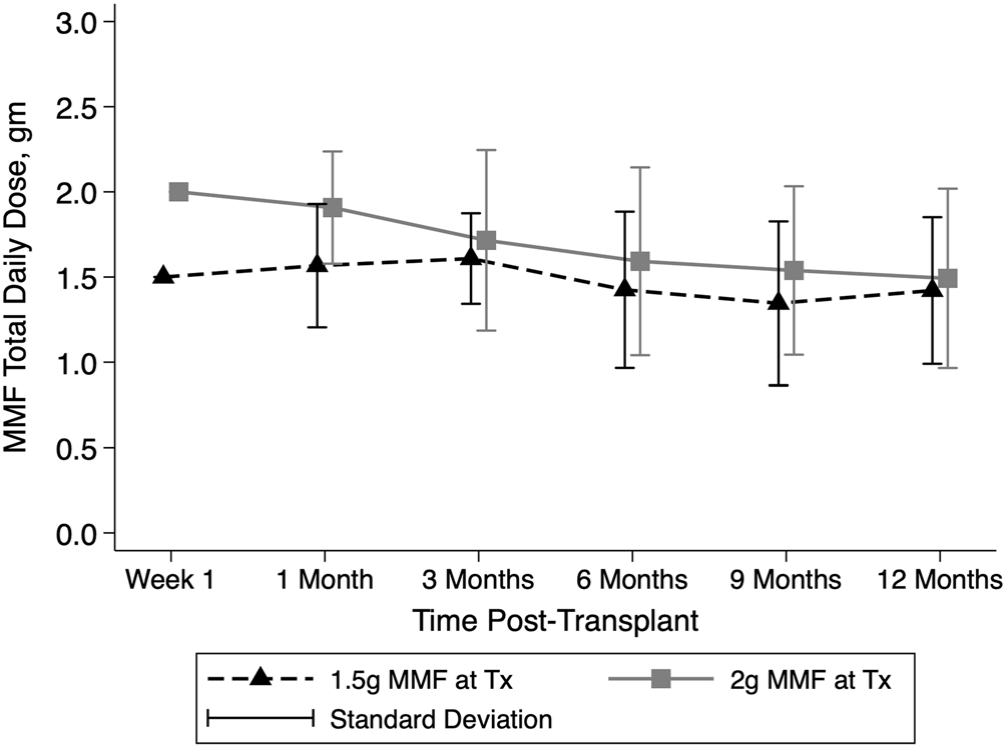
Mean mycophenolate dose by mycophenolate dose group over the initial 12 months post-transplant. *MMF* mycophenolate, *g* grams, *Tx* transplant.

During the study period, 38 de novo transplant recipients were excluded due to: initiation on cyclosporine (n = 32); initiation on an alternative immunosuppressive regimen (n = 2); and not followed up at our unit (n = 4). There were 125 patients fitting the inclusion criteria, with 60 initiated on MMF 1.5 g daily (1.5 g group) and 65 on 2 g daily (2 g group). No patients were lost to follow-up. There were no significant differences between the groups in terms of age, sex, transplant type or number, degree of HLA sensitisation or increased immunological risk, CMV donor and recipient status or tacrolimus trough levels over the first post-transplant year (Table 1, Fig. 1). There was a higher proportion of donation after circulatory death donor (DCD) transplants and a greater incidence of delayed graft function in the 2 g daily group which is likely to explain the difference in eGFR at 1 month (Tables 1, 2). While the 1.5 g group maintained a lower mean MMF dose for the first 9 months post-transplant, there was a reduction in the mean dose in the 2 g group over time such that there was no significant difference between groups by 12 months post-transplant (p = 0.8) (Fig. 2).

**Table 1:** Characteristics of patients by MMF group.

	1.5 g (n = 60)	2 g (n = 65)	p-value
Age	45.4 ± 10.8	49.1 ± 12.0	0.07
Female	26 (43%)	23 (35%)	0.59
**Transplant type**			0.31
Live donor	21 (35%)	18 (28%)	
Deceased donor	27 (45%)	38 (58%)	
SPK	12 (20%)	9 (14%)	
**Transplant number**			
1	51 (85%)	54 (83%)	0.70
2	5 (8%)	8 (12%)	
3	4 (7%)	3 (5%)	
Peak CDC PRA > 20%	10 (17%)	12 (18%)	0.79
ABOi or DSA + ve transplant	13 (21.7%)	18 (27.7%)	0.44
**Anti-CMV IgG**			0.33
D−/R−	11 (18.3%)	9 (13.8%)	
D−/R+	11 (18.3%)	21 (32.3%)	
D+/R+	23 (38.3%)	23 (35.4%)	
D+/R−	15 (25%)	12 (18.5%)	
Donor age (years)	45.3 ± 13.7	45.2 ± 16.1	0.48
Donor sex (female)	28 (46.7%)	29 (44.6%)	0.82
DCD donor	0 (0%)	9 (13.9%)	0.003
Cold ischaemic time (h)	7.3 ± 5.6	8.3 ± 5.6	0.85
Delayed graft function	7 (11.7%)	15 (23.1%)	0.09
**Tacrolimus levels (ng/mL)**			
Baseline	15.8 ± 7.5	15.1 ± 7.4	0.65
1 month	10.0 ± 2.8	9.5 ± 2.6	0.32
3 months	7.5 ± 2.0	8.1 ± 2.9	0.22
6 months	6.7 ± 1.9	7.1 ± 2.9	0.29
12 months	6.0 ± 2.0	5.4 ± 1.5	0.09

Values are n (%) or mean ± standard deviation.

*SPK* simultaneous pancreas and kidney transplant, *CDC* complement dependent cytotoxicity, *PRA* panel reactive antibody, *CMV* cytomegalomvirus, *D* donor, *R* recipient, − negative, + positive, *ABOi* blood group incompatible, *DSA* + *ve* donor specific antibody positive, *DCD* donation after circulatory death.

**Table 2:** Summary of outcomes by MMF group (univariate analysis).

	1.5 g (n = 60)	2 g (n = 65)	p-value
Acute rejection within 12 months	31 (51.7%)	25 (38.5%)	0.14
Leukopaenia within 12 months	21 (35%)	32 (49.2%)	0.06
Admissions for infection	17 (28.3%)	15 (23.1%)	0.50
CMV viraemia	3 (5%)	4 (6.2%)	0.78
BK viraemia	6 (10%)	18 (27.7%)	0.01
eGFR*			
1 month	63.9 ± 24.9	54.9 ± 23.2	0.03
12 months	64.0 ± 22.7	61.0 ± 21.6	0.47
2-years	64.0 ± 23.6	63.2 ± 22.6	0.84
3-years	61.3 ± 21.9	62.8 ± 23.3	0.81
4-years	61.1 ± 22.0	60.5 ± 21.1	0.81
5-years	60.8 ± 24.7	61.1 ± 22.7	0.97
6-years	61.9 ± 24.1	62.7 ± 23.7	0.91
7-years	62.7 ± 22.4	61.7 ± 24.0	0.94

All outcomes are to 12 months post-transplantation except eGFR.

*CMV* cytomegalovirus, *eGFR* estimated glomerular filtration rate.

*Number per group (MMF 1.5 g/2 g)—1 month (60/65), 12 months (60/65), 24 months (60/64), 36 months (59/61), 48 months (59/61), 60 months (57/60), 72 months (54/59), 84 months (49/58).

### Leukopaenia

Leukopaenia occurred in 42.4% (53 of 125) of the overall study population. Neutropaenia occurred in 49 (92.5%) of the leukopaenic patients and was not seen in any non-leukopaenic patient. The neutropaenia was mild (1000–2000 cells/mm^3^) in 14 patients, moderate (500–1000 cells/mm^3^) in 17 patients and severe (< 500 cells/mm^3^) in 18 patients. The clinical response to leukopaenia was to withhold or reduce the dose of mycophenolate, valganciclovir and sulphamethoxazole/trimethoprim in most patients (69%, 66% and 67% respectively). In the remainder, cell counts were observed without a change to medications. Colony stimulating factors were not routinely used but were reserved for patients with febrile leukopaenia. The median duration of leukopaenia was generally brief (21 days, IQR 13–39 days) while in rare cases it persisted for several months (Supplemental Fig. S1).

**Figure 3:**
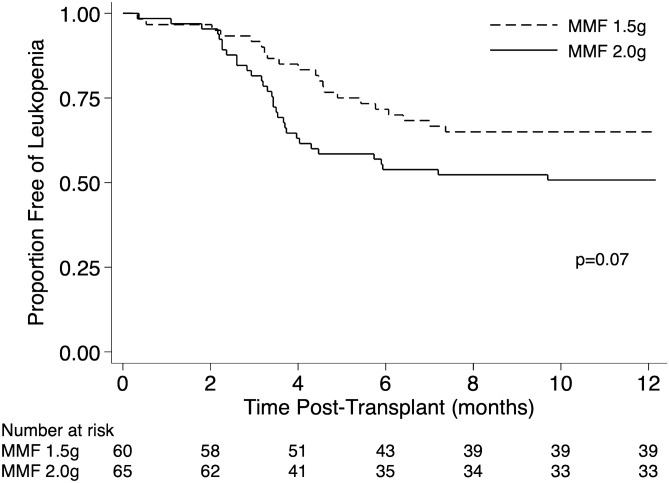
Kaplan Meier curve for leukopaenia in the initial 12 months post-transplant by mycophenolate dose group. A higher proportion of patients in the 2gm daily MMF group developed leukopaenia in the first 12 months post-transplant. The unadjusted difference was not significantly different by the log-rank test (p = 0.07). *MMF* mycophenolate, *g* grams, *Tx* transplant.

In the first post-transplant year, leukopaenia occurred more frequently in the MMF 2 g group (32 of 65, 49%) compared to the 1.5 g group (21 of 60, 35%). The unadjusted result was of borderline significance on univariate analysis (p = 0.07) (Table 2, Fig. 3). However, after adjustment for recipient age, sex, rejection episodes and CMV IgG donor positive to recipient negative, the initial MMF dose of 2 g daily (vs. 1.5 g) was associated with an increased risk of leukopaenia (HR 1.80, 95% CI 1.02 to 3.18, p = 0.04) (Table 3). Age, sex and rejection were not associated with the development of leukopaenia.

**Table 3:** Multivariable Cox regression of factors associated with the development of leukopaenia in the initial 12 months post-transplant.

	HR	95% CI	p-value
Age (per 10-year increase)	0.93	0.73–1.18	0.55
Female (vs. male)	0.66	0.37–1.18	0.16
Mycophenolate 2 g (vs. 1.5 g)	1.80	1.02–3.18	0.04
Rejection (vs. no rejection)	1.06	0.60–1.86	0.84
**CMV IgG D+ /R− **			
0 to 4 months post transplant	0.47	0.17–1.34	0.16
4 to 12 months post transplant	9.60	3.56–25.85	< 0.001

*HR* hazard ratio, *CI* confidence interval, *ref* reference group, *CMV* cytomegalovirus, *R* recipient, − negative, *D* donor, + positive.

**Figure 4:**
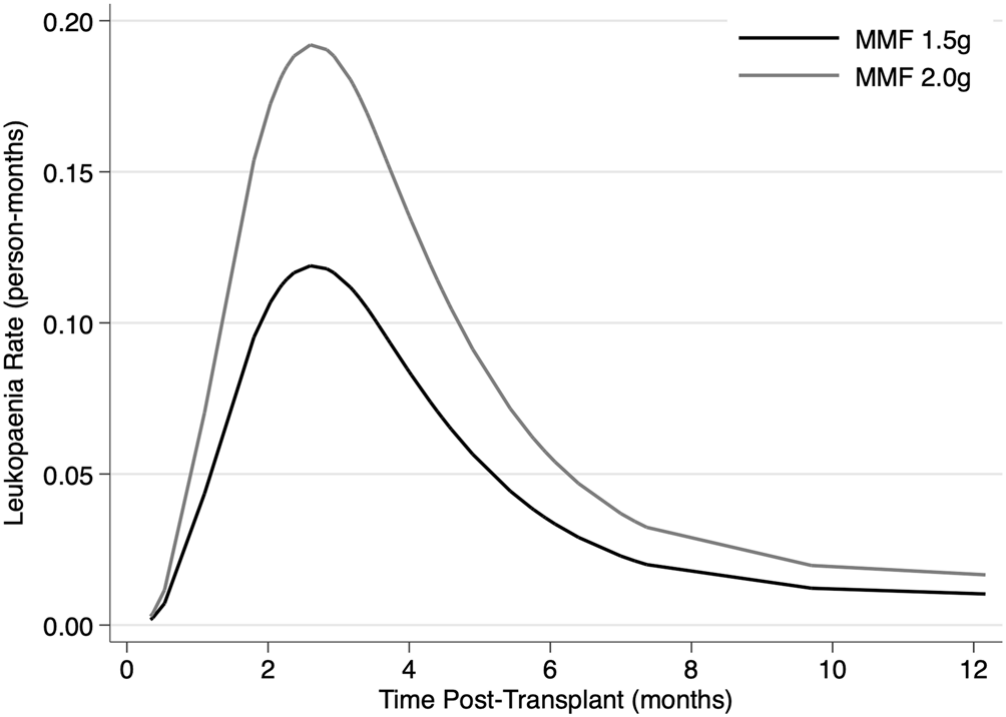
Leukopaenia rate by mycophenolate mofetil (MMF) dose group over the initial 12 months post-transplant. Using data derived from the included patients, smoothed curves were generated to illustrate leukopaenia hazard rates for the average study participant (47-year-old male without rejection), by MMF group. The incidence of leukopaenia is increased in patients taking MMF 2 g (grey lines) relative to 1.5 g (black lines). The peak incidence was at approximately 3 months post-transplant.

Figure 4 illustrates the smoothed hazard rate of leukopaenia by MMF group for the average study participant without rejection. The incidence of leukopaenia peaked between 2- and 4-months post-transplant with the incidence higher in patients in the MMF 2 gm group.

In the first 12-months post-transplant CMV viraemia occurred more frequently in patients who developed leukopaenia relative to those who did not (11.3% vs 1.4%, p = 0.02). However, in all but one case, leukopaenia was detected prior to the development of CMV viraemia so CMV viraemia was not considered causative of leukopaenia in the majority of cases. The incidence of BK viraemia was similar in patients with or without leukopaenia (15% vs. 22%, p = 0.3). Patients who developed leukopaenia did not require more frequent hospital admissions for infection than those who did not (26% vs 25%, p = 0.9). Leukopaenia was not associated with reduced renal function over the first 7 post-transplant years (p = 0.15) (Supplemental Fig. S2).

### Rejection

Any form of rejection (including borderline and subclinical) developed in 44.8% of the overall study population within the first 12 months post-transplant. This comprised antibody mediated rejection (AMR) (73.7%), cellular rejection (17.5%) and mixed rejection (8.8%). AMR was detected in the first 30 days post-transplant in the majority (78.5%) of cases, with 45.2% of these cases occurring in patients undergoing a DSA positive or ABO incompatible transplant.

**Figure 5:**
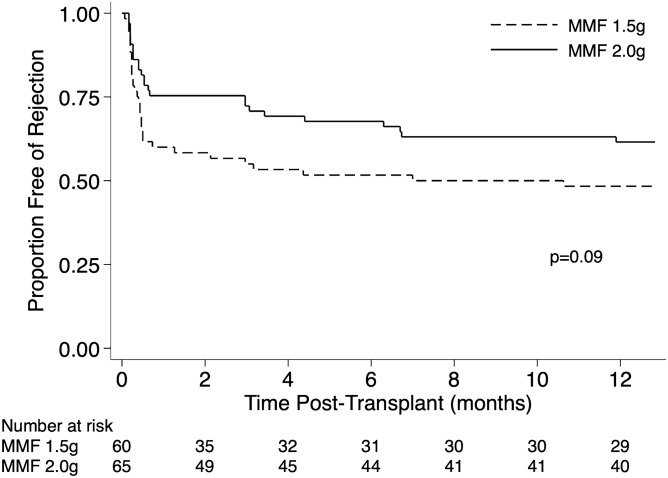
Kaplan Meier curve for rejection in the initial 12 months post-transplant by mycophenolate (MMF) dose group. A higher proportion of patients in the 1.5 g daily MMF group developed rejection in the first 12 months post-transplant. The unadjusted difference was not significantly different by the log-rank test (p = 0.09).

In the first post-transplant year, rejection episodes occurred more frequently in the MMF 1.5 g group (31 of 60, 51.7%) compared to the 2 g group (25 of 65, 38.5%). The unadjusted result was not statistically significant (p = 0.09) (Fig. 5). However, the initial MMF dose of 2 g daily (vs 1.5 g) was significantly associated with half the risk of developing rejection (HR 0.48, 95% CI 0.27 to 0.84, p = 0.01) on multivariable analysis after adjustment for recipient age, sex, leukopaenia, transplant number and increased immunological risk transplant (DSA positive or ABO incompatible) (Table 4). Increased immunological risk was associated with a fourfold increased risk of rejection (HR 4.08, 95% CI 2.08 to 8.00, p < 0.001). The development of rejection in this cohort was not associated with reduced renal function over the first 7 post-transplant years (p = 0.15) (Supplemental Fig. S3).

**Table 4:** Multivariable Cox regression of factors associated with the development of rejection in the initial 12 months post-transplant.

	HR	95% CI	p-value
Age (per 10-year increase)	1.01	0.78–1.29	0.95
Female (vs. male)	0.52	0.29–0.95	0.03
Mycophenolate 2 g (vs. 1.5 g)	0.48	0.27–0.84	0.01
Leukopaenia (vs. no leukopaenia)	0.82	0.45–1.50	0.51
**Transplant number**			
1	Ref	−	−
2	1.14	0.50–2.58	0.75
3	1.25	0.43–3.59	0.68
ABOi or DSA + ve transplant	4.08	2.08–8.00	< 0.001

*HR* hazard ratio, *CI* confidence interval, *ref* reference group, *PPRA* peak complement dependent cytotoxicity panel reactive antibody, *ABOi* blood group incompatible; *DSA* + *ve* donor specific antibody positive.

### Renal function, patient and graft survival by MMF group

**Figure 6:**
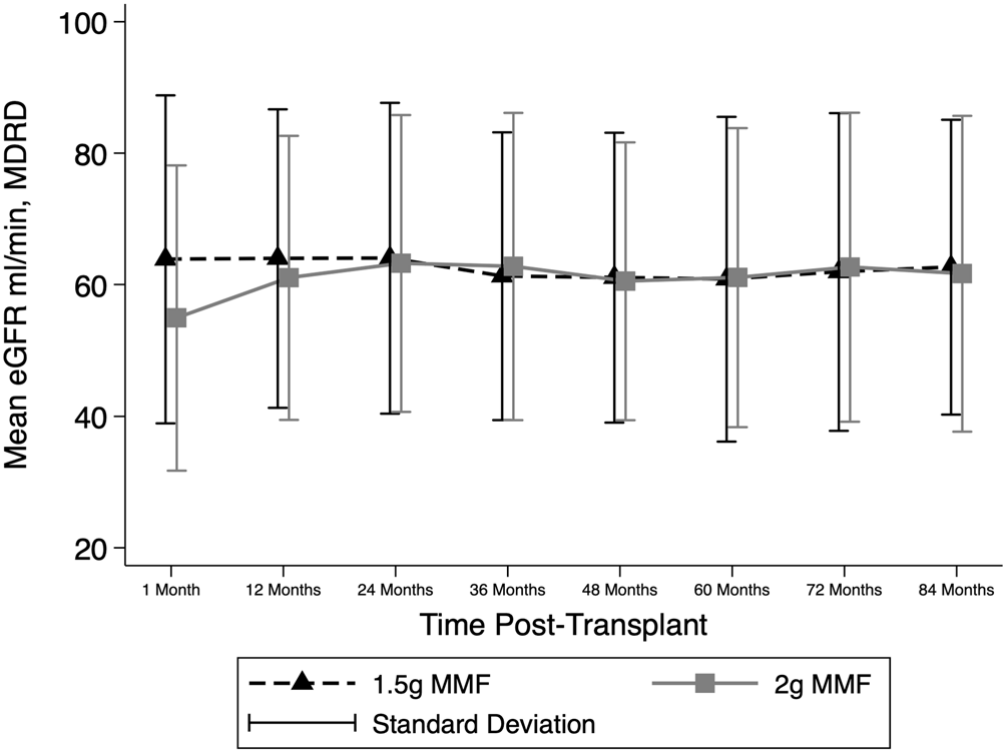
Renal function (eGFR) over the initial 7 years post-transplant by mycophenolate (MMF) dose group. eGFR was stable after the first 12 months post-transplantation and was not different between MMF groups.

Renal function (eGFR) did not differ between groups at 7-years post-transplant (p = 0.94) (Table 2, Fig. 6). The presented analyses use the CKD-Epi formula. Analyses using the MDRD 4 variable equation and the Mayo Clinic quadratic equation also showed equivalent eGFR at 7-years post-transplant. There have been 17 grafts lost and 8 deaths in the first 7 post-transplant years without a difference between groups (p > 0.17 for both). There were no significant differences in the proportion of patients who developed CMV viraemia or required hospitalisation for any infection, by group, however there was a higher incidence of BK viraemia in patients in the 2 g group relative to the 1.5 g group (27.6% vs. 10%, p = 0.01) (Table 2) but not BK viral associated nephropathy.

## Discussion

The key findings of this study are that tacrolimus treated renal transplant recipients receiving 2 g compared to 1.5 g of MMF had significantly more leukopaenia but less rejection. While these findings are somewhat intuitive, this is the first study to compare these MMF regimens in tacrolimus treated patients. We were also interested in examining the frequency of these complications in a real-world patient group and to assess the long-term impact of the initial MMF dose on renal function. The starting dose of mycophenolate had no effect on renal function at 7 years of follow-up. Leukopaenia was an extremely common early complication but was not associated with increased graft loss, rejection or poor renal function. Similarly, rejection was common, with MMF 1.5 g daily and increased immunological risk transplantation being the major risk factors detected. Rejection did not have a detrimental impact on renal function to 7 years follow-up.

MMF was introduced into the anti-rejection armamentarium in the early 1990’s^3^. The starting dose of 2 g daily has been carried forward to tacrolimus-treated patients from studies conducted in cyclosporine-treated patients^4–6^ with the practice cemented by the landmark Symphony study^1^, however given that MMF exposure is substantially higher with tacrolimus than cyclosporine it may be that a lower initial dose of MMF is sufficient. This has been examined to some extent by only a limited number of studies^18^. Those comparing 1 g daily with 2 g daily in tacrolimus-treated patients have described reduced rejection rates with the higher dose^7–10^, however this is not a universal finding with the lower dose being equivalent or superior in other studies^19,20^. Higher MMF doses are associated with higher mean area under the concentration–time curve (AUC) exposure which is associated with reduced early rejection^21,22^. However higher dosing strategies also result in an increased proportion of patients above target AUC ranges with possible additional toxicities^23,24^. Furthermore, while higher doses are associated with higher AUCs there is no data confirming that this leads to a benefit in terms of superior patient or graft survival or long-term graft function.

Fixed dosing for MMF is common practice and while there has been a move to the use of concentration controlled dosing its utility remains uncertain in tacrolimus treated patients receiving an induction agent and therefore cannot be confidently recommended by current evidence^18,22,25^. AUC measurements are cumbersome, costly, invasive and require repetition over time making them unattractive to patients and reducing their application^26^. Regardless of the use of AUC monitoring or not, a starting dose of MMF must still be selected and deriving a minimum starting dose of MMF guided by clinically relevant outcomes would be of considerable benefit in order to limit adverse events while preserving efficacy.

Although not universally accepted, it appears that 1 g of MMF daily is an insufficient starting dose to limit rejection in tacrolimus-treated patients, however evidence concerning the difference in efficacy between 1.5 and 2 g daily is lacking. Increasing the starting dose to 3 g daily results in no clear additional benefit over 2 g daily suggesting a ceiling effect^23^. Without clarity that this occurs at 2 g, it is possible that the ceiling is even lower. A single study examining enteric coated mycophenolate sodium randomised starting doses of 1440 mg vs 1080 mg in a Chinese population and demonstrated a reduced mean AUC with the lower dose but no significant differences in rejection rates or adverse events to 6 months post-transplant^19^.

Our study demonstrates that the initial doses of MMF of 1.5 g or 2 g results in no difference in graft survival or renal function out to 7 years post-transplantation suggesting that these starting doses are equally effective in the long-term. However, the initial eGFR was lower in the MMF 2 g daily group due to an increased number of DCD recipients in that cohort with delayed graft function. It is possible that this initial disadvantage may have masked a benefit of the 2 g daily dose on long-term eGFR and larger studies are needed to examine this endpoint further. There were, however significant effects of the initial dose on this studies’ primary endpoints of the development of leukopaenia and rejection within the first 12 months post-transplant. The difference in mean MMF doses between groups disappeared by 12 months suggesting that the higher dose was not tolerated by many in the 2 g daily group as there was no intention of protocolised reduction prior to 12 months. So that even with the intention of maintaining 2 g daily until 12 months in this group, most required an earlier reduction in dose. This phenomenon has also been described by Miller et al. in a 2 g daily cohort who received a mean of 1.5 g daily by 6 months due to gastrointestinal side-effects^9^. In our study an increased incidence of BK viraemia was observed in the 2 g daily group which also leads to a mycophenolate dose reduction in our centre.

Leukopaenia was a very common finding in this study and its development was significantly associated with the higher MMF dose. Previous studies have reported leukopaenia rates of 18–36% by 12 months with differences between studies likely to relate to factors such as the use of concomitant medications such as trimethoprim/sulphamethoxazole and antiviral prophylaxis^8–10,19–21,27,28^. The peak onset of leukopaenia was at 3 months post-transplant, coinciding with protocolised prednisolone reduction and hence a reduced drive to neutrophil margination. In most cases leukopaenia was relatively brief and easily managed by withholding or reducing MMF and other marrow suppressive drugs such as antiviral prophylaxis. Despite being associated with CMV viraemia, hospitalisation for serious infection was not more common in patients with leukopaenia. Patients with leukopaenia did not have a deterioration in their renal function and contrary to some previous reports we observed no association between leukopaenia and rejection^29,30^. This may relate to our reduction rather than cessation practice for MMF in the first instance. Therefore, leukopaenia is likely to have led to an increased need for blood tests, monitoring, drug dose adjustment and therefore inconvenience and health care expenses but not inferior clinical outcomes.

Rejection was also common in our patient group and occurred more frequently in the MMF 1.5 g group. The association between initial MMF dose and rejection was substantial and statistically robust, despite adjustment for the immunological risk of the transplant, which provides confidence in the validity of this finding. We included in this study, all suitable patients including those at high immunological risk. The use of surveillance biopsies in the first 7–14 days for high immunological risk patients and at 3 and 12 months in all patients, along with the inclusion of subclinical rejection in the outcome measure is likely to have resulted in a higher incidence of rejection and particularly AMR, than those described in most clinical trials. Additionally, we used an interleukin-2 receptor antagonist for induction therapy in our patients. These agents are associated with higher rejection rates than those seen with anti-thymocyte immunoglobulin, particularly in higher immunological risk transplants^31^. In general, most cases of rejection were mild and responded to treatment. Consequently, rejection was not associated with inferior graft function at 7 years follow-up in this population.

The limitations of this study include the retrospective nature of data collection, the lack of randomisation of the MMF starting dose and a smaller than optimal number of included patients which will have limited the study’s statistical power. In particular, this study was not powered to detect a difference in graft survival as it was a secondary outcome, hence a firm conclusion about MMF dose efficacy on graft survival cannot be drawn.

## Conclusions

This study demonstrates that there is clinical equipoise between a starting dose of 1.5 g or 2 g daily of MMF when used with tacrolimus. Pharmacokinetic data in this dose range is lacking and may provide useful additional information, however larger long-term randomised studies comparing clinical outcomes are the only way to determine whether such differences are of significance. Currently we recommend a MMF starting dose of 2 g daily for most renal transplant recipients to curb the incidence of rejection. Further examination of the utility of early dose reductions guided by AUC measurements or clinical features such as BK viraemia will be undertaken.

## Supplementary information

Supplementary Information.

## Abbreviations

AUC: Area under the curve
CMV: Cytomegalovirus
HLA: Human leukocyte antigen
HR: Hazard ratio
MMF: Mycophenolate mofetil
MPA: Mycophenolic acid
PRA: Panel reactive antibody
DCD: Donation after circulatory death
