# Supplementary material for: Initial mycophenolate dose in tacrolimus treated renal transplant recipients, a cohort study comparing leukopaenia, rejection and long-term graft function
Source: Sci Rep. 2020 Nov 9;10:19379. doi: 10.1038/s41598-020-76379-6 (PMC7653942; doi:10.1038/s41598-020-76379-6)
Supplement: Supplementary file 1 — Supplementary Information. [file 41598_2020_76379_MOESM1_ESM.pdf]

Title Page

Title:

Initial mycophenolate dose in tacrolimus treated renal transplant recipients, a cohort study comparing leukopaenia, rejection and long-term graft function

Authors:

Vatsa Dave[1] MBChB, Kevan R. Polkinghorne[1,2,3] MBChB PhD, Khai Gene Leong[1,2] MBBS, John Kanellis[1,2] MBBS PhD, William R. Mulley[1,2] B.Med PhD

Affiliations:

1. Department of Nephrology, Monash Medical Centre, Clayton, Victoria, Australia 3168

2. Centre for Inflammatory Diseases, Department of Medicine, Monash University, Clayton, Victoria, Australia 3168

3. Department of Epidemiology and Preventive Medicine, Monash University, Prahran, Victoria, Australia

Authors Contributions:

Vatsa Dave – Participated in: research design; writing of the paper and performance of the research.

Kevan R. Polkinghorne - Participated in: research design; writing of the paper and performance of the research.

Khai Gene Leong - Participated in: writing of the paper and performance of the research.

John Kanellis - Participated in: research design and writing of the paper.

William R. Mulley - Participated in: research design; writing of the paper and performance of the research.

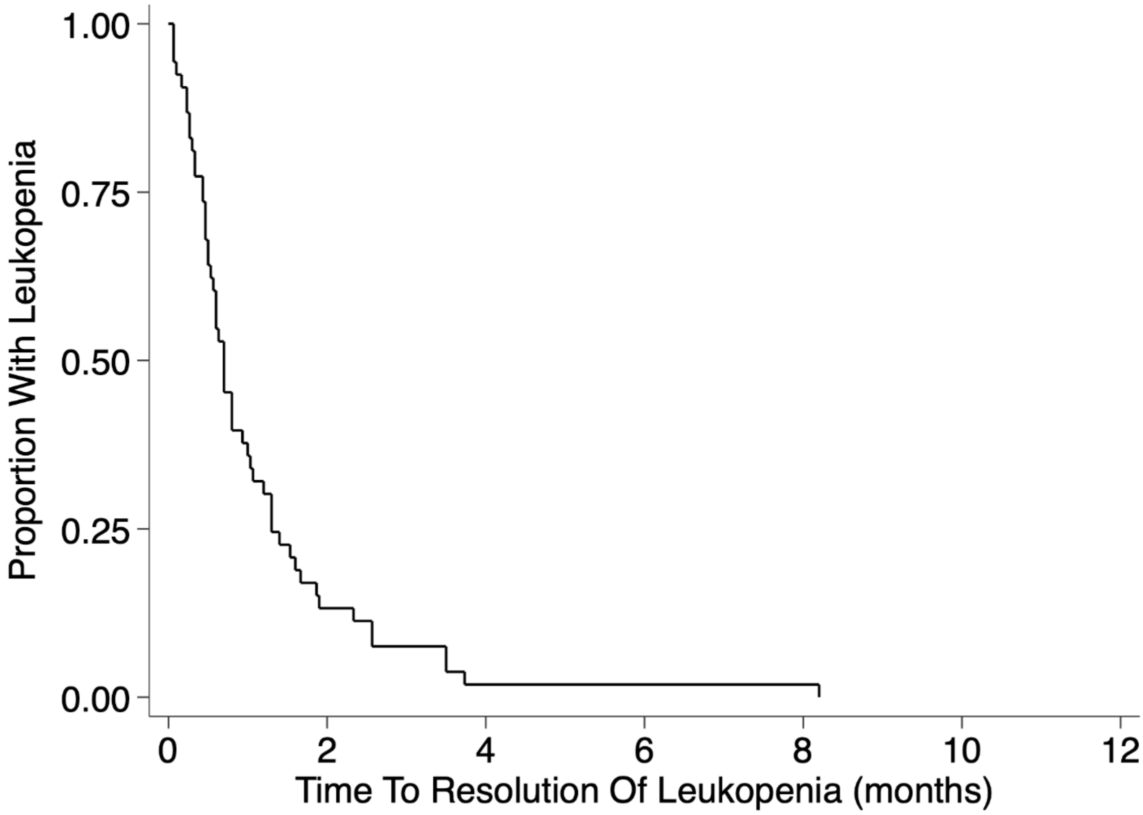

Supplemental Figure 1 – Time to resolution of Leukopaenia

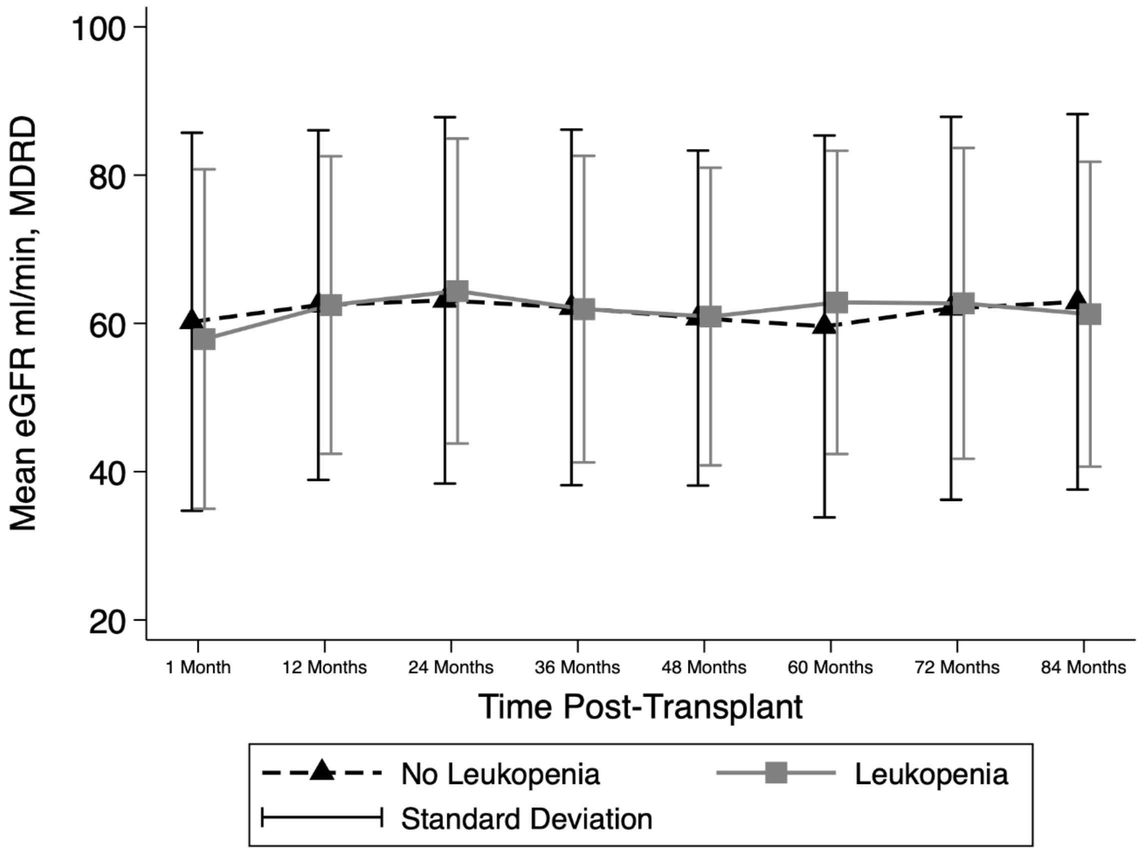

Supplemental Figure 2. Renal function (mean eGFR) over the initial 7 post-transplant years comparing patients with and without leukopaenia.

Abbreviations: eGFR = estimated glomerular filtration rate; MDRD = modified diet in renal disease formula.

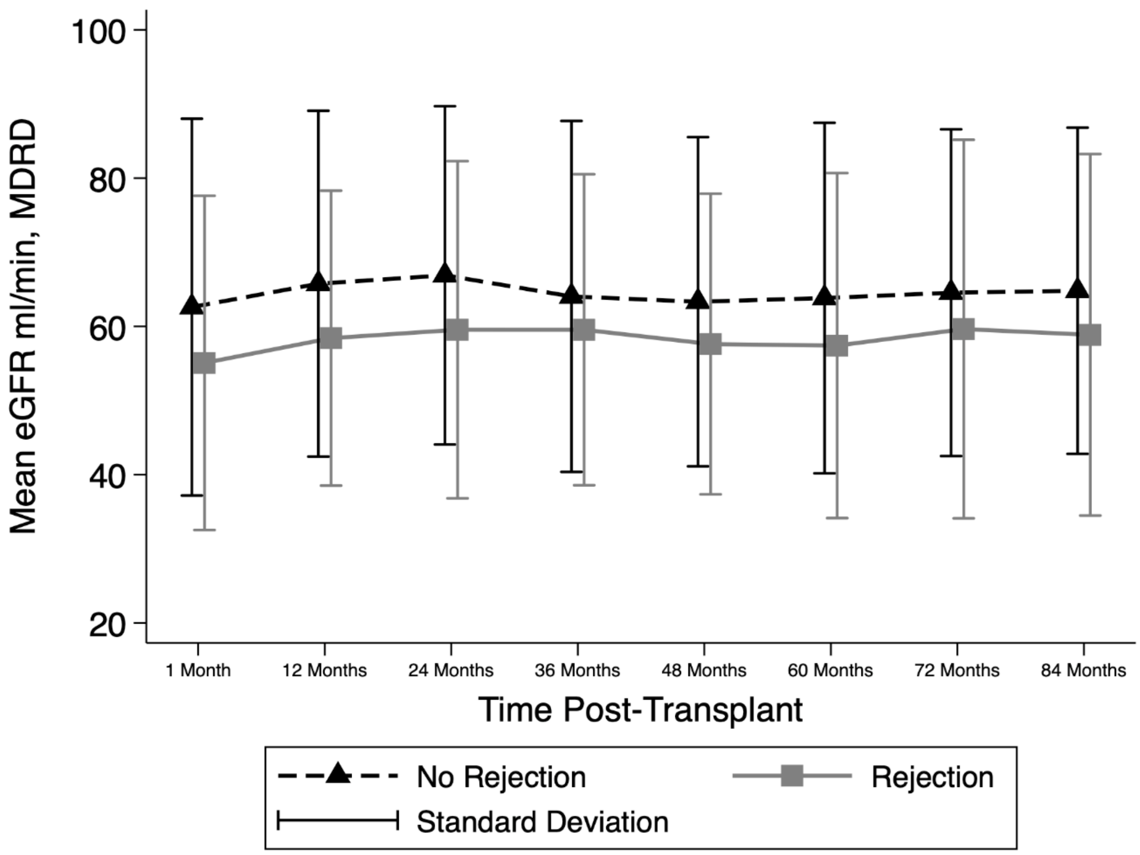

Supplemental Figure 3. Renal function (mean eGFR) over the initial 7 post-transplant years comparing patients with and without rejection.

Abbreviations: eGFR = estimated glomerular filtration rate; MDRD = modified diet in renal disease formula.
